# Therapeutic Work as a Facilitator for Return to Paid Work in Cancer Survivors

**DOI:** 10.1007/s10926-016-9641-6

**Published:** 2016-04-27

**Authors:** M. P. van Egmond, S. F. A. Duijts, P. van Muijen, A. J. van der Beek, J. R. Anema

**Affiliations:** 1 Department of Public and Occupational Health, EMGO + Institute for Health and Care Research, VU University Medical Center, Amsterdam, The Netherlands; 2 Dutch Research Center for Insurance Medicine, AMC-UMCG-UWV-VUmc, Amsterdam, The Netherlands; 3 Department of Psychosocial Research and Epidemiology, The Netherlands Cancer Institute, Amsterdam, The Netherlands; 4 Dutch Social Security Agency, Amsterdam, The Netherlands

**Keywords:** Cancer survivors, Employment, Longitudinal studies, Sick leave, Work

## Abstract

*Purpose* The increase of flexible employment in European labour markets has contributed to workers’ risk of job loss. For sick-listed workers with chronic illnesses, such as cancer, and especially those without an employment contract, participation in therapeutic work may be an important step towards paid employment. The purpose of this study was to determine the role of therapeutic employment as facilitator for return to paid work, in a cohort of sick-listed cancer survivors (CSs) with and without an employment contract. *Methods* In this longitudinal study, data were used from a cohort of Dutch CSs (N = 192), who applied for disability benefits after 2 years of sick leave. The primary outcome measure was return to paid work after 1 year. Logistic regression analysis was applied. *Results* Of the participating CSs (mean age 50.7 years, 33 % male), 69 % had an employment contract at baseline. CSs without an employment contract participated significantly less in therapeutic work (*p* < 0.001) and were less likely to return to paid work after 1 year (*p* = 0.001), than those with a contract. Participation in therapeutic work significantly increased the chance of return to paid work after 1 year (OR 6.97; 95 % CI 2.94–16.51), adjusted for age, gender, level of work disability and having an employment contract. *Conclusions* Participation in therapeutic work could be an important facilitator for return to paid work in sick-listed CSs. The effectiveness of therapeutic work as a means to return to paid employment for sick-listed workers should be studied in an experimental setting.

## Introduction

Over the past decades, new employment arrangements have emerged in the European labour market [1–3]. That is, across countries, there has been a shift from permanent employment to flexible employment, e.g., fixed-term employment contracts or temporary agency work [2]. Currently, between 8 and 33 % of workers in European countries have a flexible employment contract. To illustrate, 1,120,000 workers in the Netherlands worked in flexible employment in 2012, which is an increase of 30 % compared to 2005 [4].

Several studies have demonstrated the negative impact that flexible employment may have on workers’ health and job security [5–8]. Specifically, workers with a flexible employment contract may have poorer self-rated health, and experience higher levels of stress, fatigue and an inferior degree of mental health, compared to workers with a permanent employment contract [6, 9, 10]. Further, workers with a flexible employment contract, by definition, have no long-term job security and receive less commitment from the employer, compared to workers with a permanent employment contract [9, 11]. Especially in case of chronic illnesses, e.g., cardiovascular or respiratory diseases, diabetes or cancer, workers in flexible employment are vulnerable for job loss [12, 13].

Sick-listed workers with job loss experience more obstacles with regard to return to work (RTW) compared to sick-listed workers who still have an employment contract [14, 15]. In a recent qualitative study, workers without an employment contract, who were diagnosed with cancer, were interviewed on perceived barriers and facilitators for RTW [16]. Cancer survivors (CSs) in this study reported that participation in sheltered forms of employment, such as therapeutic work, was desirable as preparation for return to paid work. Therapeutic work involves, e.g., a gradual buildup of workload and working hours, a consistent level of RTW support, and flexibility in job demands and working hours [17]. Moreover, in therapeutic work, an employer is actively involved in the workers’ buildup process. However, opportunities for therapeutic work have diminished over the years, which may have a negative impact on the RTW of workers with cancer or another chronic condition [16]. This may be particularly true for sick-listed workers without an employment contract, as they have fewer means of RTW support than workers who still have an employment contract [18, 19].

So far, the role of therapeutic work as a step in preparation for return to paid work, has not been studied in workers with chronic illnesses, such as cancer. It is important to study if participation in therapeutic work increases the chance of return to paid work in sick-listed CSs, especially given the expected increase in CSs of working age [20]. Further, considering the increase in flexible employment, it is relevant to explore potential differences in participation in therapeutic work, between workers with and without an employment contract. Therefore, in this study, data from a national cohort of CSs in the Netherlands were used to explore the role of therapeutic employment as a facilitator for return to paid work, in workers with and without an employment contract.

## Methods

### Design

For this longitudinal study, baseline (T0) and 1-year follow-up data (T1) were used from a prospective cohort of CSs, who had been on sick leave for 2 years, and who applied for a disability benefit at the Social Security Agency (SSA) in the Netherlands [21]. In the cohort study, data were obtained from participants through questionnaires and the SSA registries. Given the fact that CSs in the cohort were assessed for work disability shortly after baseline, the outcome of CSs’ work disability assessment at the SSA was included in our analyses as a potential confounding factor. A detailed description of the study procedures of the cohort study has been published previously [21]. The cohort study was approved by the Medical Ethics Committee of the VU University Medical Center, Amsterdam, The Netherlands.

### Study Population and Procedures

All CSs who applied for a disability benefit after 2 years of sick leave, who were between 18 and 64 years old, and who had a confirmed diagnosis of cancer, were invited to participate. CSs were excluded in case of: receiving active chemotherapy and/or radiotherapy treatment, application for work disability benefits due to a somatic or psychiatric disorder other than cancer, application of a revision of a previous work disability assessment, history of self-employment and history of working in a sheltered workplace.

From July 2011 to February 2012, potentially eligible CSs were identified weekly, using a search query in the registries at the SSA headquarters. Potential participants received an information package that included an information flyer, a baseline questionnaire, and an informed consent form. CSs who returned the questionnaire and informed consent form, received a gift voucher. CSs who participated at baseline, received a follow-up questionnaire after 1 year.

For the current study, a subset of CSs who participated in the cohort was selected. To be included in this subset, CSs were selected if they were not working in paid employment at baseline, and if they were not permanently and fully disabled for work (based on the outcome of the SSA’s work disability assessment).

### Measurements

The primary outcome measure of the current study was RTW (yes; no), which was defined as return to any type of paid employment after 1 year follow-up. The independent variable in this study was participation in therapeutic work (yes; no) at baseline. The following variables were taken into account as potential confounders in the analyses: age (in years), gender (male; female), level of education (no education/primary school/lower vocational education; secondary school; vocational education/upper secondary school; upper vocational education/university), marital status (single; married; living together with partner, children and/or others; divorced/widowed), ethnicity (Dutch; non-Dutch), outcome of the work disability assessment (0–35, 35–80, 80–100 % temporary disabled, calculated as a percentage of wage loss, and categorized in accordance with the Dutch social security legislation), unemployment before start of sick leave, and employment contract during sick leave (having an employment contract; not having an employment contract).

### Statistical Analyses

*T* tests and Chi square tests were used to describe and evaluate differences in characteristics, and proportions of participation in therapeutic work and return to paid work 1 year later, between CSs with an employment contract and CSs without an employment contract. The crude association between the independent variable, i.e., participation in therapeutic work at baseline, and the dependent variable, i.e., return to paid work after 1 year, was studied using logistic regression analysis. A backward entry strategy was then used to evaluate the possibility of joint confounding, i.e., confounding by factors that individually do not lead to confounding, but when combined do lead to confounding. Using this specific strategy, all potential confounders were entered into the model, after which potential confounders were randomly removed from the model. If the removal of a variable caused a change in the regression coefficient of the independent variable of >10 %, the change was considered meaningful and the variable remained in the model. The crude association was adjusted for age and gender regardless of any relevant confounding, as this allows for comparison between previous studies. SPSS 22.0 was applied to conduct the analyses [22].

## Results

### Study Population

Of the 484 participants in the original national cohort, 192 CSs were eligible for the current study. Of these, 39 % participated in therapeutic work at baseline. The mean age was 50.7 years, 33 % was male, and 70 % was married. Over 96 % of the study population had the Dutch nationality, more than two-third (69 %) had an employment contract, and 31 % did not have an employment contract at baseline. The average total of working years prior to sick leave was 25.5 years. The characteristics of the study population are presented in Table 1.

**Table 1:** Characteristics of CSs with and without an employment at baseline

Variable	Categories	Total group (N = 192)	CSs (contract); N = 132)	CSs (no contract; N = 60)	*p* value*
		Mean (SD)	Mean (SD)	Mean (SD)	
Age (years)		50.7 (8.0)	50.4 (7.6)	51.4 (9.0)	0.444
Years working before sick leave		25.5 (11.2)	24.7 (10.8)	27.2 (11.8)	0.162

		N (%)^a^	N (%)^a^	N (%)^a^	
Gender	Male	63 (32.8)	38 (28.8)	25 (41.7)	0.078
	Female	129 (67.2)	94 (71.2)	35 (58.3)	
Level of education	None/primary/lower vocational education	56 (29.2)	38 (28.8)	18 (30.0)	0.432
	Secondary school	35 (18.2)	24 (18.2)	24 (18.2)	
	Vocational education/upper secondary school	56 (29.2)	35 (26.5)	35 (26.5)	
	Upper vocational education/university	45 (23.4)	36 (26.5)	35 (26.5)	
Principal wage earner	No	87 (45.5)	62 (47.3)	25 (41.7)	0.466
	Yes	104 (54.5)	69 (52.7)	35 (58.3)	
Marital status	Unmarried	17 (8.9)	8 (6.1)	9 (15)	0.211
	Married	135 (70.3)	95 (72.0)	40 (66.7)	
	Living together	14 (7.3)	11 (8.3)	3 (5.0)	
	Divorced/widowed	26 (13.5)	18 (13.6)	8 (13.3)	
Having children	No	50 (26.0)	33 (25)	17 (28.3)	0.626
	Yes	142 (74.0)	99 (75)	43 (71.7)	
Ethnicity	Dutch	185 (96.4)	127 (96.2)	58 (96.7)	0.876
	Non-Dutch	7 (3.6)	5 (3.8)	2 (3.3)	
Tumor type	Breast	86 (44.8)	57 (43.2)	29 (48.3)	0.464
	Urinary tract	14 (7.3)	10 (7.6)	4 (6.7)	
	Urogenital male	6 (3.1)	4 (3.0)	2 (3.3)	
	Urogenital female	7 (3.6)	4 (3.0)	3 (5.0)	
	Respiratory tract	8 (4.2)	8 (6.1)	0 (0.0)	
	Digestive system	25 (13.0)	15 (11.4)	10 (16.7)	
	Head and neck	10 (5.2)	9 (6.8)	1 (1.7)	
	Hematological	29 (15.1)	19 (14.4)	10 (16.7)	
	Central nervous system	2 (1.0)	2 (1.5)	0 (0.0)	
	Other type of cancer	5 (2.6)	4 (3.0)	1 (1.7)	
Metastasized cancer	No	109 (57.7)	73 (56.6)	36 (60.0)	0.907
	Yes lymph nodes	70 (37.0)	49 (38.0)	21 (35.0)	
	Yes, distant	10 (5.3)	7 (5.4)	3 (5.0)	
Treatment modalities	Surgery	147 (76.6)	97 (73.5)	50 (83.3)	0.135
	Radiotherapy	114 (59.4)	79 (59.8)	35 (58.3)	0.843
	Chemotherapy	143 (74.5)	100 (75.8)	43 (71.7)	0.547
	Hormone therapy	56 (29.2)	38 (28.8)	18 (30.0)	0.864
	Immunotherapy	15 (7.8)	11 (8.3)	4 (6.7)	0.690
	No treatment	1 (0.5)	1 (0.8)	0 (0.0)	0.499
Declared free of disease by physician	No	48 (25.8)	27 (20.8)	21 (37.5)	0.057
	Yes	84 (45.2)	63 (48.5)	21 (37.5)	
	Do not know	54 (29.0)	40 (30.8)	14 (25.0)	
Comorbidity	No	108 (56.3)	77 (58.3)	31 (51.7)	0.388
	Yes	84 (43.8)	55 (41.7)	29 (48.3)	
Work disability assessment (temporary disabled)	<35 %	41 (21.4)	22 (16.7)	19 (31.7)	0.062
	35–80 %	57 (29.7)	41 (31.1)	16 (26.7)	
	80–100 %	94 (49.0)	69 (52.3)	25 (41.7)	
Participation in therapeutic work	No	117 (60.9)	64 (48.5)	53 (88.3)	<0.001
	Yes	75 (39.1)	68 (51.5)	7 (11.7)	
Return to paid work after 1 year	No	139 (72)	86 (65)	53 (88)	0.001
	Yes	53 (28)	46 (35)	7 (12)	
Type of sector previous job	Blue collar	73 (43.5)	56 (42.4)	17 (47.2)	0.006
	White collar	45 (26.8)	29 (22.0)	16 (44.4)	
	Civil servant	14 (8.3)	13 (9.8)	1 (2.8)	
	Health care worker	36 (21.4)	34 (25.8)	2 (5.6)	
Shift work previous job	No	103 (53.6)	80 (60.6)	23 (38.3)	<0.001
	Yes	65 (33.9)	52 (39.4)	13 (21.7)	
	Not applicable (unemployed before sick leave)	24 (12.5)	0 (0)	24 (40.0)	
Managerial tasks previous job	No	137 (71.4)	106 (81.5)	31 (51.7)	<0.001
	Yes	29 (15.1)	24 (18.5)	5 (8.3)	
	Not applicable (unemployed before sick leave)	24 (12.5)	0 (0.0)	24 (40.0)	
Previous job demands	Psychological and physical	82 (42.9)	68 (51.9)	14 (23.3)	<0.001
	Mainly psychological	44 (23.0)	29 (22.1)	15 (25.0)	
	Mainly physical	41 (21.5)	34 (26.0)	7 (11.7)	
	Not applicable (unemployed before sick leave)	24 (12.6)	0 (0.0)	24 (40.0)	

* *p* values are the result of *T* tests and Chi square tests for differences in characteristics between CSs with and without an employment contract

^a^The calculated totals of numbers and percentages per variable may approach or exceed 100 % because of missing values, the option to provide multiple answers, or rounding differences

### Therapeutic Work, Return to Paid Work and Having an Employment Contract

A significant crude association between participation in therapeutic work and RTW 1 year later was found. In these unadjusted analyses, CSs who participated in therapeutic work, had a significantly higher odds of RTW 1 year later, compared to CSs who did not participate in therapeutic work [odds ratio (OR) 12.26; 95 % confidence interval (CI) 5.68–26.50]. Of the potential confounders, the outcome of the work disability assessment and having an employment contract, had a significant influence on the association between therapeutic work and RTW. That is, CSs in the lower categories of work disability (i.e., 0–35, 35–80 % temporary disabled) had a significantly higher chance of RTW (*p* < 0.001) than CSs in the highest category of work disability (i.e., 80–100 % temporary disabled. Further, CSs without an employment contract participated significantly less often in therapeutic work (*p* < 0.001), and were less likely to RTW after 1 year (*p* = 0.001), than those with an employment contract. The association between therapeutic work and return to paid work 1 year later, was thus adjusted for age, gender, outcome of the work disability assessment and having an employment contract (OR 6.97; 95 % CI 2.94–16.51). The results of the logistic regression analyses are presented in Table 2.

**Table 2:** Association between participation in therapeutic work at baseline and RTW 1 year later

	Crude model			Adjusted model 1			Final model		
	OR	95 % CI	*p* ^†^	OR	95 % CI	*p* ^†^	OR	95 % CI	*p* ^†^
Therapeutic work^a^	12.26	5.68–26.50	<0.001	9.70	4.33–21.70	<0.001	6.97	2.94–16.51	<0.001
Age				0.98	0.93–1.03	0.368	0.97	0.92–1.03	0.311
Gender				1.48	0.60–3.66	0.392	1.42	0.57–3.55	0.457
0 to <35 % disabled^b^				5.16	1.85–14.42	0.002	6.50	2.19–19.32	0.001
35 to <80 % disabled^b^				3.92	1.48–10.39	0.006	4.33	1.63–11.52	0.003
No employment contract^c^							0.38	0.13–1.12	0.078

*OR* odds ratio

^†^ *p* value

^a^Compared to the reference category “not participating in therapeutic work”

^b^Compared to “80–100 % work disabled”

^c^Compared to the reference group “having an employment contract”

## Discussion

### Main Findings

The main finding of this study is that CS who participated in therapeutic work at baseline had a highly increased chance of return to paid work 1 year later, compared to those who did not perform therapeutic work. Furthermore, CSs without an employment contract participated significantly less in therapeutic work, and were less likely to return to paid employment after 1 year, compared to CSs with an employment contract.

### Interpretation of Findings

In this study, CSs who participated in therapeutic work at baseline, were far more likely to return to paid employment within 1 year follow-up. So far, no studies have particularly reported on the role of therapeutic work as a facilitator for RTW. Still, there are other studies that also describe RTW facilitators, somewhat comparable to therapeutic work [23, 24]. That is, therapeutic work is characterized as sheltered work, with fewer obligations, fewer stress-inducing activities, and more room for accommodation to the workers’ needs, than regular employment [17]. Several of these characteristics of therapeutic employment have been identified as individual RTW facilitators by other studies in workers with cancer and other chronic illnesses [23, 24]. For example, two large reviews by Mehnert et al. [25] and Spelten et al. [26] found that, amongst other factors, perceived employer accommodation, counseling, and miscellaneous training services at work, were important facilitators for RTW in CSs. Further, from a chronic illnesses perspective, Boot et al. conducted a large mixed-methods study on RTW in older workers with chronic illnesses. Although no quantitative association between work-related factors and RTW were found, qualitatively, workers reported that psychosocial resources at work, e.g., support from colleagues and the employer, were important facilitators for RTW [27]. In a related study, psychosocial resources at work were found to be predictive of RTW in older workers with chronic disease, although not in workers without chronic disease [28]. Further, studies in CSs and workers with chronic illnesses have also reported on the crucial role that employers have with regard to RTW, i.e., providing support and a sense of value, taking care of practical arrangements and communication to colleagues on behalf of the worker [29–31]. As therapeutic work combines several of these RTW facilitators, i.e., psychosocial resources at work, gradual buildup of workload and support from an employer, into a single working arrangement, this may explain why CSs in our study, who participated in therapeutic work at baseline, were more successful in returning to paid employment within 1 year, than workers who did not participate in therapeutic work.

Further, in this study, the association between therapeutic work and return to paid work was significantly influenced by the outcome of CSs’ work disability assessment short after baseline. This seems plausible, as multiple studies across workers have demonstrated that higher levels of work disability decrease the chance of RTW [32, 33]. It should be mentioned that both impaired health, as well as corresponding financial incentives in the form of disability benefits in Western social security systems, may contribute to the negative association between higher levels of work disability and decreased chance of RTW [11].

This study also revealed that CSs with an employment contract participated significantly more often in therapeutic work at baseline, and logically, were significantly more often at work in paid employment after 1 year, compared to CSs without an employment contract. The difference in participation in therapeutic work between CSs with and without an employment contract may be explained by access to therapeutic work, as well as motivation for (therapeutic) RTW. First, it should be considered that opportunities to participate in therapeutic work during sick leave are often provided by an employer, either because the employer is obliged to do so by law, or because of an employer’s commitment to the worker. Two previous studies in employers of workers with breast cancer and chronic musculoskeletal pain demonstrated that employers can be committed and willing to invest in their employee [34, 35]. For CSs without an employment contract, logically, there is no employer who is legally obliged, or intrinsically motivated, to provide RTW support in the form of therapeutic work. As a result, CSs who have an employment contract are, at least in the Netherlands, by definition more likely to have access to therapeutic work, compared to CSs who are no longer employed. For the latter group, it is theoretically still possible that a previous employer would offer them therapeutic employment, but this is rarely the case. After all, the previous employer did not renew the employment contract in the first place. It is therefore plausible that both the legal context of employment arrangements, as well as the personal attitude of employers, influences CSs’ access to therapeutic employment.

Finally, motivation may play a role in the decision to participate in therapeutic work, as therapeutic work is often a form of preparation for return to paid work [36]. Potentially, CSs who participated in therapeutic work in this study, were more motivated to RTW, or were physically or mentally better prepared for RTW, than those who were not participating in therapeutic work. Previous studies in CSs, as well as studies in workers with other chronic illnesses, have shown that a better health status, and corresponding higher levels of work ability, may reduce the duration of sick leave and increases the likelihood of RTW [33, 37–39]. Further, it has been widely documented that chronic illnesses, including cancer, may have a significant impact on the meaning that is attributed to work [40, 41]. That is, after facing a life-threatening disease (return to) work may become less important, while family or hobbies may become more important [42, 43]. Possibly, a change in the meaning of work, combined with the experience of job loss, has an impact on the extent to which CSs in this study were motivated to participate in therapeutic work and to RTW [16]. However, such conclusions should be drawn cautiously, as other studies have also reported that work may remain important for CSs during and after treatment [36, 42].

### Strengths and Limitations

The main strength of this study is that data were used from a national cohort of CSs in the Netherlands. There are also several limitations that should be mentioned. First, the sample of CSs in this study was relatively small. Also, because of substantial differences between the groups in this study, there is a possibility of residual confounding. Both these factors limit the generalizability of our results. Further, we know from previous studies that between 84 and 94 % CSs returned to work within 24 months of sick leave [25]. As this study included CSs who did not yet return to paid work within 24 months, it is plausible that our results apply to CSs who, compared to (the majority of) CSs as described in other studies, struggle with RTW more, or in different ways. Also, as this was a longitudinal cohort study, no conclusive evidence with regard to causality can be drawn from our results. Finally, our results should be interpreted in the context of a national social security system, and translation of these results to non-Western countries should be done cautiously.

### Implications for Future Research and Practice

The findings of this study indicate that there may be a beneficial relationship between therapeutic work and return to paid work in workers with cancer. Further, we recommend that researchers investigate if RTW in CSs may be facilitated by providing access to therapeutic work or comparable forms of sheltered employment in a randomized controlled trial. Further, the extent to which our results can be extrapolated to larger populations of CSs and workers with other chronic illnesses, should be studied. Moreover, as flexible employment keeps increasing in Western countries [44], it is vital that practitioners and policymakers explore opportunities for access to therapeutic work and psychosocial resources, compatible with these new employment arrangements. The key to enhancing labour market participation of workers with chronic illnesses, could be for governments and institutions to offer or subsidize therapeutic employment arrangements, a responsibility which presently lies almost exclusively in the hands of employers.

## Conclusion

Participation in therapeutic work could be an important facilitator for return to paid work in sick-listed CSs. The effectiveness of therapeutic work as a means to return to paid employment should be studied in experimental settings. If effective, policymakers may pave the way to therapeutic work or similar constructs of sheltered work for CSs, particularly for those without an employment contract, in order to prepare for RTW in paid employment.

## Abbreviations

CS(s): Cancer survivor(s)
RTW: Return to work
SSA: Social security agency
